# AIM2 inflammasome-derived IL-1β induces postoperative ileus in mice

**DOI:** 10.1038/s41598-019-46968-1

**Published:** 2019-07-22

**Authors:** Kristof Johannes Hupa, Kathy Stein, Ralf Schneider, Mariola Lysson, Bianca Schneiker, Veit Hornung, Eicke Latz, Yoichiro Iwakura, Jörg C. Kalff, Sven Wehner

**Affiliations:** 1 Department of Surgery, University of Bonn, Bonn, Germany; 2 Institute of Experimental Medicine, University of Bonn, Bonn, Germany; 3 Institute of Innate Immunity, University of Bonn, Bonn, Germany; 4 Research Institute for Biomedical Sciences, Tokyo University of Science, Chiba, 278-0022 Japan; 5 Present Address: Gene Center and Department of Biochemistry, Ludwig-Maximilians-Universität München, Munich, 81377 Germany

**Keywords:** Motility disorders, Innate immunity

## Abstract

Postoperative ileus (POI) is an intestinal dysmotility frequently occurring after abdominal surgery. An orchestrated neuroimmune response within the muscularis externa (ME) involves activation of resident macrophages, enteric glia and infiltration of blood-derived leukocytes. Interleukin-1 receptor type-I (IL1R1) signalling on enteric glia has been shown to be involved in POI development. Herein we investigated the distinct role of the IL1R1 ligands interleukin (IL) -1α and IL-1β and focused on the mechanism of IL-1β production. IL-1α and IL-1β deficient mice were protected from POI. Bone-marrow transplantation studies indicated that IL-1α originated from radio-resistant cells while IL-1β was released from the radio-sensitive infiltrating leukocytes. Mouse strains deficient in inflammasome formation identified the absent in melanoma 2 (AIM2) inflammasome to be crucial for IL-1β production in POI. Mechanistically, antibiotic-treated mice revealed a prominent role of the microbiome in IL-1β production. Our study provides new insights into distinct roles of IL-1α and IL-1β signalling during POI. While IL-1α release is most likely an immediate passive response to the surgical trauma, IL-1β production depends on AIM2 inflammasome formation and the microbiome. Selective interaction in this pathway might be a promising target to prevent POI in surgical patients.

## Introduction

POI is a common complication of abdominal surgery and is characterized by a transient impairment of the propulsive gastrointestinal motility^1,2^. Patients suffer from discomfort and increased morbidity which leads to prolonged hospital stay and an increased medico-economic burden^3^. Previous work indicates that POI pathophysiology comprises a complex inflammatory response to the surgical intestinal manipulation (IM) leading to the activation of resident macrophages and enteric glia^4,5^. A central immune pathway in POI involves IL1R1 signalling in enteric glia^5^. Upon IL-1 ligation, enteric glia releases macrophage chemoattractant protein -1 (MCP-1) and IL-6 which both contribute to POI pathogenesis^5–7^, IL-1α and IL-1β are ligands of the IL1R1 with identical biological activities^8^. Nevertheless, different biological responses of both were described e.g. recruitment of different subsets of myeloid cells during sterile inflammation^9^ and non-redundant properties during acute intestinal inflammation^10^. IL-1α is constitutively present in many non-hematopoietic cells^11–13^ and exhibits bioactivity at IL1R1 even in its immature uncleaved pro-form^14^ which can be released as an early danger associated molecular pattern (DAMP)^11,15^. In contrast, immature uncleaved IL-1β is not able to induce IL1R1 signalling^16^ but requires cleavage by caspase-1 before it can be secreted^17,18^ as a bioactive IL1R1 ligand. Caspase-1 is part of a multienzyme complex called inflammasome that requires the adaptor protein ASC (apoptosis-associated speck-like protein containing a caspase activation and recruitment domain) and specific cytosolic receptors for assembly^17^. The most prominent cytosolic receptor is NLRP3 (NACHT, LRR and PYD domains-containing protein 3) which is able to sense numerous molecules including crystals, fatty acids and bacterial toxins. Other sensors are NLRC4 (NLR family CARD domain-containing protein 4) or AIM2 which sense bacterial flagellin and the bacterial type-III secretion apparatus^19^ or cytosolic dsDNA^20^, respectively. The latter can originate from bacteria or viruses^21^ or from endogenous origins i.e. from stress-damaged nuclei or mitochondria^22^.

In health, IL-1α is produced by a variety of cells but basal tissue levels of bioactive IL-1β are hardly detectable. Recently, we described a cleavage of caspase-1 and IL-1β release during POI^5^ but the precise mechanisms of IL-1β release remained unclear. In the present study we investigated the individual roles of IL-1α and IL-1β during POI and focussed on the identification of molecular mechanisms which lead to IL-1β release during POI.

## Results

### IL-1α and IL-1β deficiency protects mice from POI

**Figure 1:**
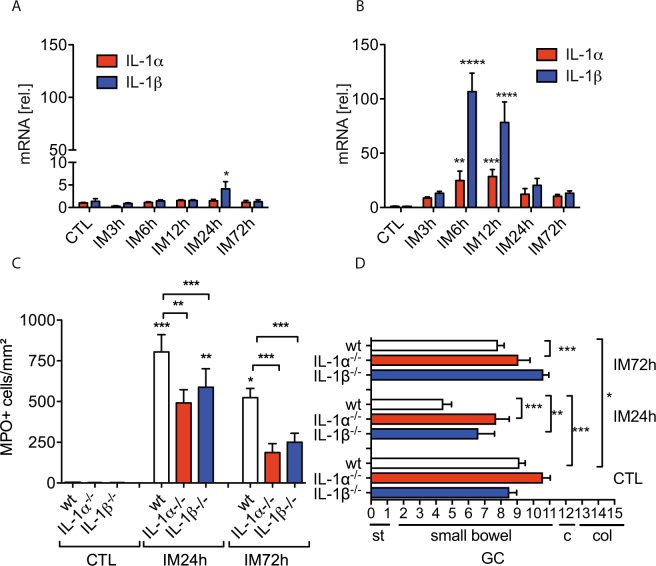
IL-1α and IL-1β deficient mice are protected from POI. (**A**) Wt mice underwent IM. Gene expression of IL-1α and IL-1β was analysed in small bowel mucosa (**A**) and ME (**B**). Statistical analysis: one-way ANOVA and Bonferroni test. (n = 5 per group). Wt, IL-1α^−/−^ and IL-1β^−/−^ mice underwent IM. (**C**) Subsequently, myeloperoxidase^+^ (MPO^+^) cells were counted in ME whole mount specimen and (**D**) *in vivo* gastrointestinal motility. Statistical analysis: two-way ANOVA and Bonferroni test. n = 3 for CTLs, n_wt IM24h_ = 11, n_IL-1_α^−/−^_IM 24h_ = 7, n_IL-1_β^−/−^_IM24h_ = 9, n_wt IM72h_ = 8, n_IL-1_α^−/−^_IM72h_ = 5, n_IL-1_β^−/−^_IM72h_ = 5. CTL = untreated animals, GC = geometric centre, st = stomach, c = cecum, col = colon.

First, we analysed IL-1α and IL-1β gene expression at several postoperative time points in the ME and mucosa. Interestingly, we found that neither IL-1α nor IL-1β, with exception of a slightly increase of IL-1β at 24 h, were induced in the intestinal mucosa up to 72 h after IM (Fig. 1A) while IL-1α/β gene expression was strongly upregulated at 6 h and 12 h in the ME (Fig. 1B). We next subjected IL-1α^−/−^ and IL-1β^−/−^ mice to IM and both demonstrated reduced MPO^+^ leukocyte infiltration into the ME (p < 0.01 for IL-1α^−/−^ and p < 0.001 for IL-1β^−/−^ mice) compared to manipulated wildtype (wt) mice 24 h after IM (Fig. 1C). MPO^+^ cell numbers did not differ between both IL-1 deficient mouse strains and remained reduced (p < 0.001) up to 72 h after IM compared to wt mice. Furthermore, the GI transit was accelerated in IL-1 deficient mice as shown by the increased geometric centres (GC) (IL-1α^−/−^ GC: 7.7 ± 2.1, p < 0.001; IL-1β^−/−^ GC: 6.6 ± 3.0, p < 0.01; wt GC: 2.5 ± 2.0) (Fig. 1D) 24 h after IM. At 72 h, the GI transit was still retarded (GC: 7.8 ± 1, p < 0.05) in wt mice while IL-1 deficient mice had already recovered from POI (IL-1α^−/−^ GC :9.1 ± 1.6; IL-1β^−/−^ GC: 10.6 ± 0.8).

**Figure 2:**
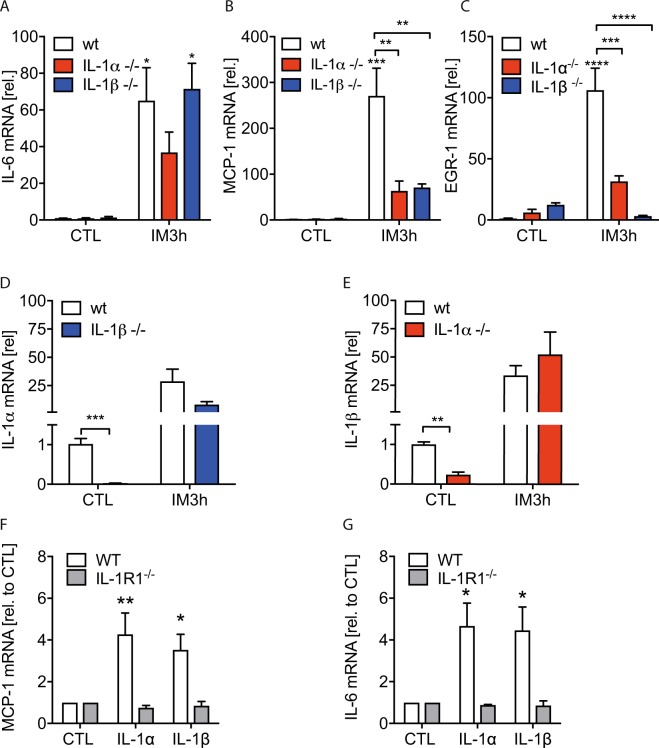
Inflammatory gene expression during POI is reduced in IL-1α and IL-1β deficient mice. Wt, IL-1α^−/−^ and IL-1β^−/−^ mice underwent IM. Subsequently, (**A**–**E**) mRNA expression of indicated genes was quantified in small bowel ME. Statistical analysis: 1-way ANOVA and Bonferroni test. (n = 3 for CTLs and n = 5 for IM per group). (**F**,**G**) Enteric glia cell cultures from wt and IL-1R1^−/−^ mice were stimulated with IL-1α or IL-1β for 24 h and MCP-1 or IL-6 mRNA levels were analysed. Statistical analyses: two-way ANOVA followed by Bonferroni posthoc test. (n = 5 per group). CTL = (**A**–**E**) untreated animals or (**F**,**G**) vehicle-treated cells.

We next analysed whether IL-1α^−/−^ or IL-1β^−/−^ mice exhibit an altered inflammatory response in the early phase of POI. IL-6 gene expression was not induced in IL-1α^−/−^ in contrast to wt and IL-1β^−/−^ mice while the early growth response protein 1 (EGR-1) and MCP-1 were reduced in IL-1α^−/−^ and IL-1β^−/−^ mice, 3 h after IM (Fig. 2A–C). We also checked both IL-1 deficient mouse strains for IL-1α and IL-1β expression. As expected, IL-1α or IL-1β were not transcribed in IL-1α^−/−^ or IL-1β^−/−^ mice, respectively. However, IL-1α gene expression was robustly reduced in naïve IL-1β^−/−^ mice and vice versa (Fig. 2D,E). As uncleaved pro-IL-1α is able to signal via the IL1R1, the reduced gene expression of IL-6, EGR-1 and MCP-1 in the early phase of POI is most likely mediated by the concomitant IL-1α deficiency in IL-1β deficient mice. Notably, IL-1α was regularly induced in IL-1β^−/−^ mice 3 h upon IM, indicating that the concomitant IL-1α reduction in naïve IL-1β^−/−^ mice is only present in homeostasis but not after surgery. Additionally, we tested the biological activity of both IL-1 ligands in enteric glial cells. IL-1α and IL-1β induced a comparable gene expression increase of IL-6 (p < 0.05) and MCP-1 (p < 0.05 for IL-1β and p < 0.01 for IL-1α) (Fig. 2F,G).

### IL-1α and IL-1β originate from different cell populations

**Figure 3:**
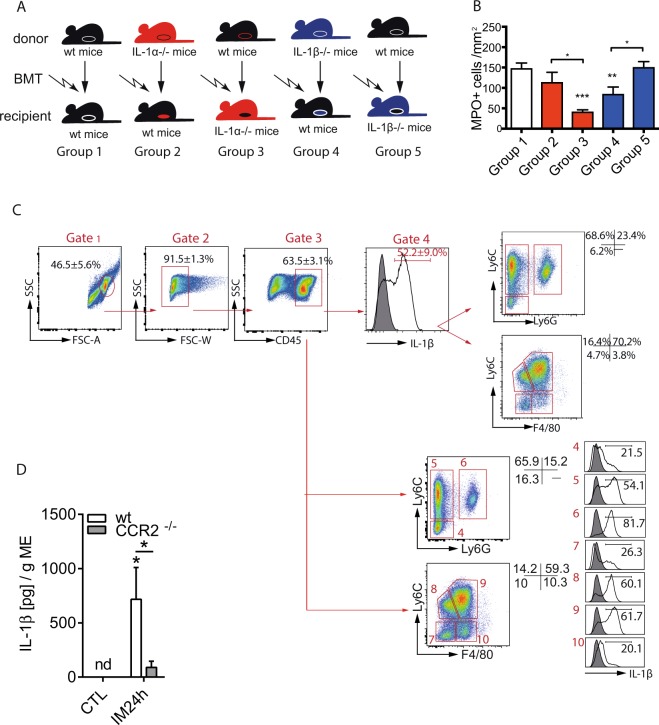
Infiltrating leukocytes are the relevant source of IL-1β during POI. Myelo-depleting irradiation and bone marrow transplantation (BMT) identified different cellular sources of IL-1α and IL-1β. (**A**) Schematic overview on the experimental groups. Irradiated recipients which received BMT are show at the bottom line and donor mice at the top line. Six weeks after BMT, all recipients underwent IM. (**B**) Myeloperoxidase^+^ (MPO^+^) cells were counted in ME whole mount specimens. Statistical analysis: 1-way ANOVA and Bonferroni test. n_group 1_ = 11, n_group 2_ = 11, n_group 3_ = 9, n_group 4_ = 10, n_group 5_ = 7. (**C**) Infiltrating CD45^+^ leukocytes where subdivided by Ly6C, Ly6G or F4/80 expression and analysed for IL-1β expression 24 h after IM. n = 3 per group (**D**) ME organ culture supernatants from wt or CCR2^−/−^ mice were analysed for IL-1β release. Statistical analysis: student’s t-test. (n = 3 for CTL, n = 5 for IM24h). CTL = untreated animals.

To identify the cellular source of IL-1α and IL-1β in the postoperative ME we generated IL-1 chimeric mice by bone marrow transplantation (BMT) which exhibit IL-1α or IL-1β deficiency either in radio-sensitive hematopoietic or radio-resistant resident tissue cells (Fig. 3A). IL-1α^−/−^ mice that received wt BMT showed reduced postoperative MPO^+^ leukocyte numbers (group 3, p < 0.05; 52 ± 15 cells/mm^2^) compared to wildtype mice reconstituted with wt BM (group 1, 176 ± 107 cells/mm^2^) (Fig. 3A). Wt mice that received IL-1α^−/−^ BMT had elevated leukocyte numbers (group 2, 114 ± 82 cells/mm^2^) and did not differ from group 1. Strikingly, we observed the opposite effect in IL-1β chimeric mice. WT mice that received IL-1β^−/−^ BMT showed a reduced leukocyte influx (group 4, p < 0.05; 85 ± 55cells/mm^2^) while IL-1β^−/−^ mice that received wt BMT demonstrated higher (group 5, p < 0.05; 151 ± 36 cells/mm^2^) cell counts. These data indicate that radio-resistant resident cells are responsible for the IL-1α mediated effects, while radio-sensitive hematopoietic cells account for the IL-1β triggered response during POI. These results support the hypothesis of IL-1α acting as a DAMP in the early phase of POI.

Next, we aimed to further characterize the IL-1β releasing cell type. Flow cytometry studies identified more than 50% of CD45^+^ leukocytes expressing IL-1β after IM and the majority of IL-1β^+^ cells (Fig. 3C, gate 4) were Ly6C^+^Ly6G^−^ infiltrating monocytes (68.6% ± 5.5). Ly6C^+^Ly6G^+^ neutrophils (23.4% ± 5.7) and a minor population (6.2% ± 0.2) of non-further characterised CD45^+^ cells also contributed to IL-1β production. Within the individual cell populations, 54.1% of Ly6C^+^Ly6G^−^ monocytes and 61.7% of their Ly6C^+^F4/80^+^ monocyte-derived macrophage descendants expressed IL-1β (Fig. 3C, gate 5 and 9, respectively). Interestingly, only 20.1% of the Ly6C^−^F4/80^+^ resident macrophages but 81.7% of Ly6C^+^Ly6G^+^ neutrophils (81.7%) expressed IL-1β (Fig. 3C, gate 10 and 6, respectively). Given that monocytes account for the majority of infiltrating cells during POI we hypothesized that CCR2-deficient mice, which were shown to almost completely lack infiltrating monocytes in the postoperative ME^23^, have reduced IL-1β levels during POI. Indeed, IL-1β release was reduced in CCR2^−/−^ compared to wt mice (Fig. 3D).

### IL-1β release depends on the AIM2 inflammasome

**Figure 4:**
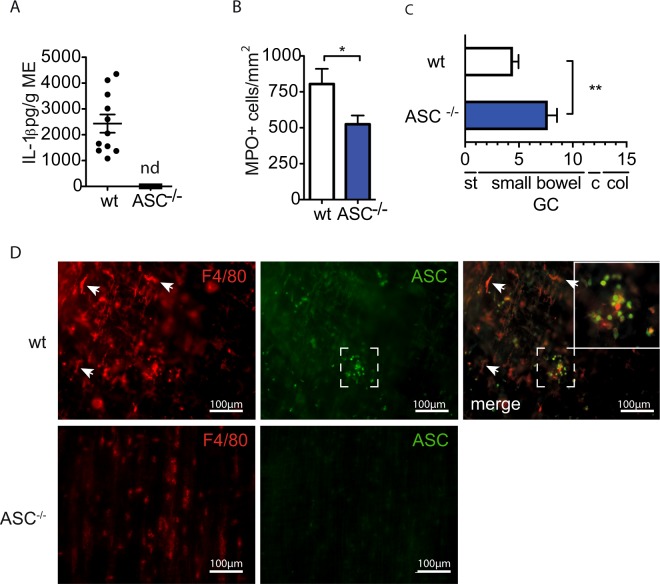
Inflammasome activation contributes to POI. Wt or ASC^−/−^ mice underwent IM and were analysed after 24h for (**A**) IL-1β release from ME organ culture supernatants by ELISA (n_wt_ = 11, n_ASC−/−_ = 5, nd = not detectable); (**B**) myeloperoxidase^+^ (MPO^+^) cells of the ME and (**C**) *in vivo* gastrointestinal motility (**C**). Statistical analyses were performed with a one-way ANOVA followed by Bonferroni posthoc test (B: n_wt_ = 6, n_ASC−/−_ = 9) (C: n_wt_ = 15, n_ASC−/−_ = 7). (**D**) Representative immunofluorescence microscopy images of F4/80 and ASC expression in ME whole mount specimens (n = 3). CTL = untreated animals, GC = geometric centre, st = stomach, c = cecum, col = colon.

**Figure 5:**
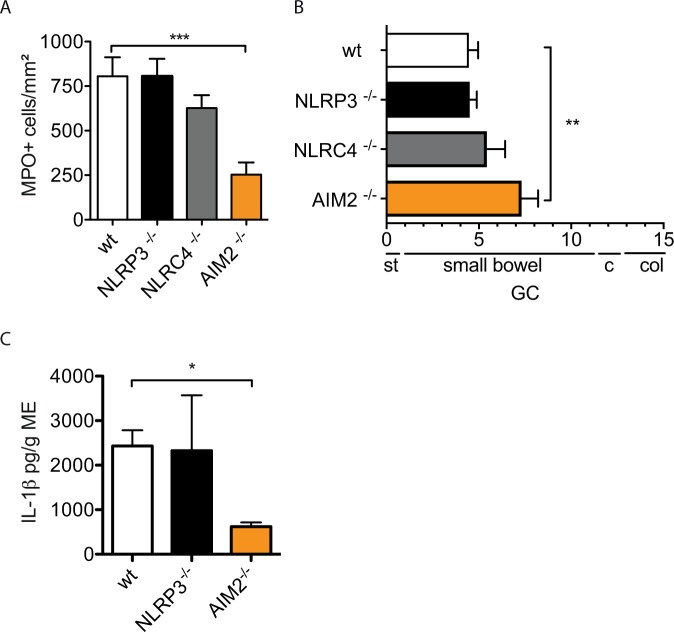
AIM2 deficiency protects mice from POI. Wt, NLRP3^−/−^, NLRC4^−/−^ and AIM2^−/−^ mice underwent IM. Analysis was performed 24h after IM. (**A**) Infiltrating myeloperoxidase (MPO)^+^ cells were counted in ME whole mount specimens. (n_wt_ = 6, n_NLRP3_^−/−^ = 5, n_NLRC4_^−/−^ = 6, n_AIM2_^−/−^ = 9). (**B**) *In vivo* gastrointestinal motility was analysed by detection of the geometric centre (GC). (**B**) (n_wt_15, n_NLRP3−/−_ = 5, n_NLRC4−/−_ = 6, n_AIM2−/−_ = 9). (**C**) IL-1β production was measured in ME organ culture supernatants by ELISA. (**C**) (n_wt_11, n_NLRP3−/−_ = 4, n_AIM2_^−/−^ = 4). Statistical analyses: 1-way ANOVA with Bonferroni test. GC = geometric centre, st = stomach, c = cecum, col = colon.

The bridging molecule ASC is essential for full inflammasome assembly and caspase-1 cleavage. Therefore we subjected ASC-deficient mice to IM which demonstrated a complete absence of postoperative IL-1β release (Fig. 4A) in postoperative ME organ cultures (wt: 1156 ± 1243 pg/g ME, ASC^−/−^: 0 ± 0 pg/g ME). Postoperative MPO^+^ leukocyte numbers were reduced (Fig. 4B) and the GI-transit was accelerated (wt GC: 4,5 ± 2.0, ASC^−/−^ GC: 7.7 ± 2.2) (Fig. 4C). Immunofluorescence microscopy identified ASC expression in round-shaped infiltrating F4/80^+^ leukocytes while stellate F4/80^+^ resident macrophages did not express ASC (Fig. 4D). These data confirmed the involvement of inflammasome activation during POI. Next, we tested NLRP3-, NRLC4- and AIM2-deficient mice for leukocyte extravasation and GI motility. In NLRP3^−/−^ (806 ± 218 cells/mm^2^) and NLRC4^−/−^ mice (626 ± 178cells/mm^2^) postoperative MPO^+^ cell numbers did not differ from wt mice (805 ± 260 cells/mm^2^) and postoperative GI transit was not improved (Fig. 5A,B, NLRP3^−/−^: GC:4.5 ± 0.9, NLRC4^−/−^: GC:5,4 ± 2.4, wt mice GC:4.5 ± 2.0). However, AIM2^−/−^ mice demonstrated reduced MPO^+^ leukocyte influx (253 ± 207cells/mm^2^) and an accelerated GI transit (GC:7,3 ± 2.6) 24 h after IM. Confirmative, ME from AIM2^−/−^ mice released less IL-1β (620 ± 185 pg/g) compared to wt mice (2432 ± 1170 pg/g) while NLRP3^−/−^ did not differ (1859 ± 2396 pg/g) (Fig. 5C) after IM.

### IL-1β release requires presence of luminal bacteria

The AIM2 inflammasome recognizes cytoplasmic double-stranded DNA (dsDNA) which can originate from endogenous or exogenous sources. Endogenous sources could be DNA release by dying or damaged cells or mislocation of nuclear or mitochondrial dsDNA upon oxidative stress. Exogenous sources could be luminal bacteria or viruses that pass the mucosal barrier upon surgical trauma and infect cells.

**Figure 6:**
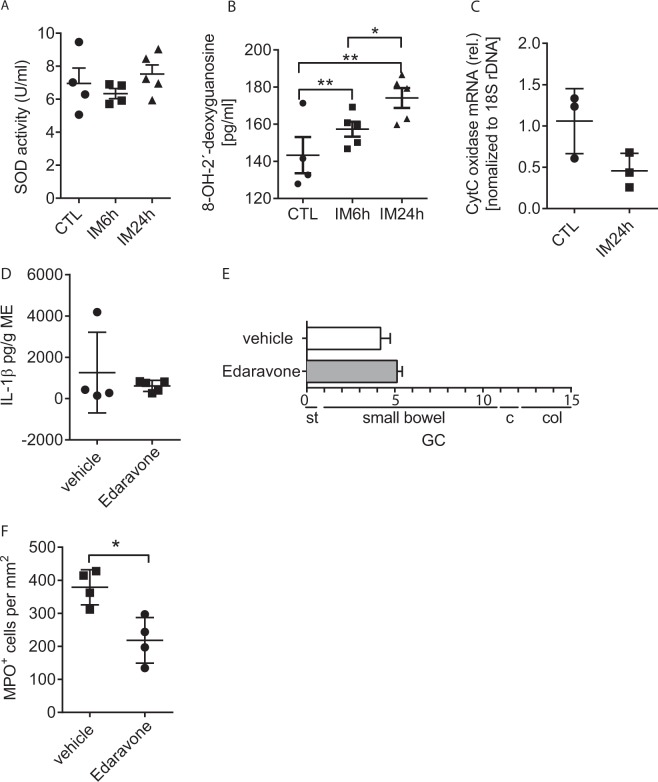
Oxidative stress is not a trigger of IL-1β release during POI. Wt mice underwent IM followed by analyses of (**A**) superoxide dismutase activity, (**B**) 8-OH-2′-deoxyguanosine levels and (**C**) cytosolic Cyt C DNA levels in the ME. Wt mice underwent antioxidant (Edaravone) or vehicle-treatment perioperatively and were analysed for (**D**) IL-1β release from ME organ cultures, (**E**) gastrointestinal motility and (**F**) numbers of myeloperoxidase (MPO)^+^ leukocytes 24h after IM. Statistical analyses: student’s t-test (**A**,**B**: n_CTL_ = 4, n_IM_ = 5; C: n = 3; **D**–**F**: n = 4,). GC = geometric centre, st = stomach, c = cecum, col = colon.

We analysed postoperative ME samples for the presence of oxidative stress but did neither detect reactive oxygen species (ROS) by dihydroethidium (DHE) nor the reactive lipid mediator 4-hydroxynonenal by immunofluorescence microscopy in the ME of naïve or intestinally manipulated mice (results not shown). Furthermore, we did not detect increased superoxide dismutase (SOD) activity (Fig. 6A) after IM but found a time-dependent increase of 8-OH-2-deoxyguanosin in the postoperative ME (Fig. 6B). This DNA modification may be a sign of oxidative stress to mitochondrial DNA and mitochondrial damage but cytosolic DNA levels of the mitochondrial gene cytochrome (Cyt) -C oxidase were not increased upon IM (Fig. 6C). As these findings did not clearly indicate whether oxidative stress is involved in POI or not, we tested if an antioxidant treatment ameliorates POI. In various models of neurological diseases, Edaravone (3-Methyl-1-phenyl-5-pyrazolon) has been shown to prevent oxidative stress and neuronal death. At a dose of 10 mg/kg bodyweight, Edaravone prevented cervical motor neuron loss^24^ in a murine amyotrophic lateral sclerosis model while in rats doses between 3–10 mg/kg with different application routes were reported to effectively reduce oxidative stress in cerebral ischemia models^25,26^. In our model we used an i.p. injection of 10 mg/kg but did not observe any effects on IL-1β release (Fig. 6D) nor on GI transit (Fig. 6E) during POI. We further analysed the effects of an Edaravone i.v. injection but observed comparable effects (Supplementary Fig. S1A,B). However, MPO^+^ leukocyte numbers were reduced in the Edaravone treated animals (Fig. 6F, Supplementary Fig. S1C). These data indicate that oxidative stress might be involved in POI pathogenesis but is not directly linked to IL-1β production.

**Figure 7:**
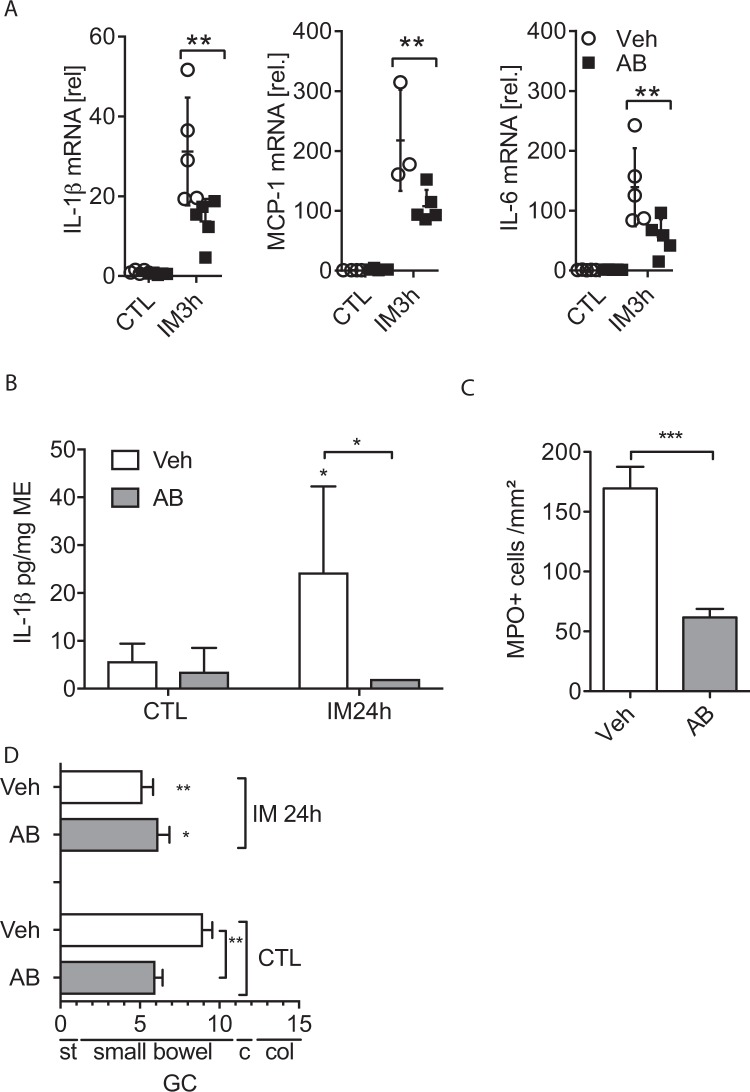
Presence of the microbiome is crucial for IL-1β release during POI. Wt mice received repetitive gavages of oral antibiotics (AB) or water (Veh) for 7 days and were subjected to IM. (**A**) mRNA expression of the indicated genes was analysed in the ME. (n = 5 per group). (**B**) IL-1β production was measured by ELISA in ME organ culture supernatants. (**C**) Myeloperoxidase MPO^+^ cell counts in the ME. (**D**) *In vivo* gastrointestinal motility. Statistical analyses: student’s t-test or one-way ANOVA with Bonferroni test (n = 5 per group). CTL = untreated animals, GC = geometric centre, st = stomach, c = cecum, col = colon.

Therefore, we next tested whether the intestinal microbiome is involved in the postoperative inflammation and IL-1β production and treated mice for 7 days with oral antibiotics before they were subjected to IM. Efficacy of the antibiotic treatment was confirmed by absence of cultivable aerobic and anaerobic bacteria in stool samples after 7 days (results not shown). We observed a reduction in IL-1β, MCP-1 and IL-6 gene expression in antibiotic-treated mice (Fig. 7A) and ME organ culture supernatants released less IL-1β protein (Fig. 7B). MPO^+^ cells were also reduced (Fig. 7C) but the gastrointestinal transit was not accelerated (Fig. 7D). Notably, non-operated antibiotic-treated mice already had a reduced transit time (p < 0.01), indicating that the motility was generally disturbed by the antibiotic treatment.

Together, our study demonstrates that IL-1 signalling is crucial for POI. While IL-1α predominantly acts in the early phase IL-1β is involved in the late phase release and depends on the AIM2 inflammasome formation and presence of the intestinal microbiome.

## Discussion

Previous work of our group demonstrated that IL1R1 induces POI in mice, a process that is hypothesized to be mediated via an IL-1 dependent release of IL-6 and MCP-1 from enteric glial cells^5^. In the present manuscript we investigated the role of IL-1α and IL-1β in the pathogenesis of POI. IL-1α- as well as IL-1β-deficiency protects mice from POI as shown by an accelerated GI transit, reduced ME leukocyte infiltration and an accelerated resolution observed 72 h after surgery. The limited induction of IL-1α and IL-1β gene expression in the ME but not in the lamina propria mucosae indicates a site-restriction of the bowel wall inflammation to the ME and this was also shown for other cytokines i.e. IL-6, IL-12 and TNF (tumor necrosis factor)^27^ in mice but also in humans^28^. IL-1α and IL-1β both target the IL1R1 and were described to exhibit the same biological activity which was also confirmed in our study wherein they induced IL-6 and MCP-1 release in enteric glial cells to the same extent. Nevertheless, IL-1α and IL-1β were shown to exhibit distinct biological activities mainly by originating from different cellular sources and at different time points in the course of disease^9,10^. Distinct roles of both cytokines can also be postulated for POI. As IL-1α is ubiquitously expressed^18^ almost every resident tissue cell type could release IL-1α upon surgical trauma. As IL-1α is bioactive in its immature precursor form^28^ and was described to act as a DAMP in sterile inflammation we conclude that stressed or dying cells of the surgically manipulated ME release IL-1α which in turn activates IL1RI in the early phase of POI. This theory is supported by several observations: First, absence of translocating bacteria in the early phase of POI indicates that onset of POI triggers a sterile inflammation^29^. Second, cleaved IL-1α is absent in the ME up to 6 h after surgical manipulation^5^. Third, IL-1α^−/−^ mice demonstrated reduced MCP-1 expression in the early postoperative phase. Fourth, we identified radioresistant cells, which are mostl likely resident tissue cells, as IL-1α source during POI. As IL-1α is also transcriptionally induced during POI^5^ and can also be cleaved from its pro-form into the mature IL-1α by calpain^30^, which is also ubiquitously expressed in mammals and many other organisms^31^, IL-1α might of course also contribute to the late phase of POI. Surprisingly, IL-1β^−/−^ mice also demonstrated reduced MCP-1 levels in the early postoperative ME. This finding was puzzling as IL-1β is not ubiquitously expressed in ME cells, its mature form is not detectable within the first 3 h after IM^5^ and release of the bioactive mature IL-1β requires inflammasome-activation^32^. However, IL-1α mRNA levels were 50-fold reduced in the ME of naïve IL-1β^−/−^ mice. Horai *et al*. also detected a 30-fold reduced basal IL-1α mRNA levels in brains of IL-1β^−/−^ mice^33^. The severe IL-1α reduction might explain the reduction of IM-induced MCP-1 in IL-1β^−/−^ mice. The other way around, IL-1β mRNA were also reduced in IL-1α^−/−^ mice but to a much lower extent (4-fold) than IL-1α was in IL-1β^−/−^ mice. This was also shown in brains by Horai *et al*.^33^. Notably, in contrast to the brain inflammation model investigated by Horai and colleagues, we observed a normal postoperative upregulation of either IL-1α or IL-1β in the ME of IL-1β- or IL-1α-deficient mice, respectively. This shows that the de novo synthesis of IL-1α in IL-1β^−/−^ mice and vice versa is not disturbed during POI.

IL-1β may be rather important in the late phase of POI what is mainly supported by the identification of infiltrating cells as its cellular source. In contrast to IL-1α-, IL-1β-deficiency in radiosensitive cells protects mice from POI and further analyses identified infiltrating F4/80^+^Ly6C^+^Ly6G^−^ monocyte-derived macrophages, their Ly6C^+^Ly6G^−^ monocyte ancestors and Ly6C^+^Ly6G^+^ neutrophils as the predominant IL-1β producing cells. The distinct reduction of IL-1β expressing cells in CCR2-deficent mice, which lack monocytes in the postoperative ME^23,34^ confirms the origin of IL-1β. As none of these cell types is present in the ME at the beginning of surgery, IL-1β is unlikely to be involved in the initial steps of POI. Another reason for a delayed role of IL-1β is the temporal aspect of its maturation which requires inflammasome assembly^32^. Caspase-1 activation, the enzyme that cleaves pro-IL-1β into its mature form has already been shown to become activated during POI but it is inactive in the naïve ME^5^. Herein we confirm that mice that are incompetent in inflammasome assembly^35^ (ASC^−/−^ mice) do not release IL-1β from the postoperative ME and do not develop POI. Interestingly, the well characterized NLRP3 inflammasome, binding multiple molecular patterns^36–38^ and the NLRC4 inflammasome, detecting bacterial flagellin and components of the salmonella type III secretion system^19^ were neither involved in the release of IL-1β nor in the postoperative motility disturbances. However, mice deficient for AIM2, a cytosolic sensor of double-stranded DNA^39^ demonstrated markedly decreased IL-1β levels and showed ameliorated leukocyte influx and POI. Presence of ASC immunoreactivity in infiltrating round shaped immune cells, which are most likely monocytes or monocyte-derived macrophages, again confirmed the finding that IL-1β majorly originates from the infiltrating but not ME resident cells. In humans, neutrophils were shown to express the full inflammasome machinery^40^ and neutrophil-derived IL-1β might therefore play a role during POI in surgical patients.

The involvement of AIM2 in POI leads to the question where the cytosolic dsDNA originates from. Our data provide evidence that luminal pathogens rather than endogenous or self dsDNA, which is released into the cytosol from the nucleus or the mitochondria during oxidative stress^41^, contribute to IL-1β release during POI. Actually, both pathways are not unlikely to play a role during POI. Oxidative stress was often observed upon surgery for example in cardiac surgery or liver surgery^42,43^. ROS production is increased upon abdominal surgery^44,45^ and increases epithelial permeability^46,47^. Increased intraoperative oxidative stress was also detected in blood samples upon open but not laparoscopic surgery^48^ and surgical stress also induces mitochondrial and nuclear DNA^49^. However, in the present study we did not find clear signs of oxidative stress during POI. Importantly, Edaravone, failed to prevent IL-1β and POI independent of its application route. On the other hand, Edaravone reduced the postoperative ME leukocyte numbers, which are the main source of IL-1β. While these data do not allow a conclusion about the involvement of oxidative stress in POI they still show that IL-1β production is not directly linked to oxidative stress in this model. It should be noted that in this experiment we observed a reduced IL-1β release from vehicle-treated animals in contrast to previous experiments in wt mice (i.e. Figs 1B and 4A) and this is also in line with the diminished numbers of infiltrating MPO^+^ cells in this group. We speculate that the fluid administration per se could reduce the postoperative immune response to some degree but obviously not strong enough to prevent POI development.

In contrast, eradication of the intestinal microbiome by antibiotics reduced IL-1β expression during POI. We also recently demonstrated that intestinal antimicrobial peptides are regulated in an IL-1R1 dependent manner in POI^29^. An impact of oral antibiotics on the length of POI was shown in two recent metanalysis in patients undergoing colorectal surgery^50,51^ and omission of oral antibiotics was found to be an independent risk factor for development of POI^3^. Nevertheless, the role of the microbiome in POI is not totally clear, as the antibiotic regime used in our study did not improve GI transit and antibiotic-treated animals demonstrated disturbed motility even in absence of the surgical procedure which was also shown by others^52^. However, IL-1β release and leukocyte numbers were strongly reduced by the antibiotics and this at least demonstrates that the microbiome is involved in IL-1β production during POI. Another putative source of dsDNA may be self-DNA loaded exosomes whose release from immune cells is augmented during chemotherapy-induced inflammation and induces the AIM2 inflammasome^53^. As inflammation during POI remains localized to the ME, exosome release, e.g. to the blood, is hardly detectable in our model and remains speculative.

The identification of the selective AIM2 pathway is of particular relevance, as inhibition of IL-1β itself may have side effects in surgical patients, e.g. on anastomotic healing, as it is critical in cutaneous wound healing^54^. Also the intervention on the central parts of inflammasome activation (i.e. caspase-1 and ASC inhibition) may also have side effects as it was shown that ASC deficiency impairs skin wound healing^54^ and caspase-1 and ASC deficiency are susceptible to DSS induced colitis and heightened intestinal permeability^55^. The intervention on specific cytosolic NOD-like receptor levels might appear more selective although it was also shown that NLRP3^−/−^ mice are more susceptible to DSS colitis^56^. As AIM2 deficient mice are more susceptible to inflammatory bowel disease^57,58^ this may also apply to an AIM2 intervention however, a role for AIM2 in anastomotic healing has never been investigated so far. However, AIM2 targeting drugs remain to be developed. Alternative molecules capable to sequester cytosolic dsDNA like p202^59^ or an IFI16 transcript with the same domain architecture as p202^60^ could also be investigated.

In summary, our results demonstrate that IL-1α and IL-1β contribute to POI. IL-1α acts as a DAMP in the initial phase of POI whereas IL-1β is released during late phase of POI in an AIM2 inflammasome- and microbiome dependent manner. Inhibition of AIM2 inflammasome activation may prevent excessive intestinal IL-1β production in surgical patients and could be a promising and selective approach to prevent POI.

## Material and Methods

### Animals

Experiments were performed using 8–12-week-old mice kept in a pathogen-free animal facility with standard rodent food and tap water ad libidum. C57/BL/6J mice served as wild type control (wt) and were purchased from Janvier, Saint Berthevin Cedex, France). Furthermore we used IL-1α^−/−^^33^, IL-1β^−/−^^33^. IL-1RI^−/−^, CCR2^−/−^ were obtained from Jackson Laboratories (Charles River, Sulzfeld, Germany). ASC^−/−^, NLRP3^−/−^, NLRC4^−/−^ and AIM-2^−/−^ were obtained from Genentech, USA. All experiments were performed in accordance to federal law for animal protection and approved by proper authorities of North-Rhine-Westfalia (Landesamt für Natur-, Umwelt- und Verbraucherschutz, LANUV).

### Bone marrow transplantation

Bone marrow cells (BM) were collected from the femur and tibia of wt, IL-1α^−/−^ or IL-1β^−/−^ donor mice. Host mice were irradiated (9 Gy) and received 1.2 × 10^7^ cells intravenously 7 h after irradiation. Five chimera groups were generated: wildtype reconstituted with wildtype BM (group 1), and IL-1α^−/−^ or IL-1β^−/−^ reconstituted with wildtype BM (group 3 and 5) or vice versa (group 2 and 4). All experiments were performed in accordance with federal law regarding animal protection and were approved by the state agency for nature, environment and consumer protection (LANUV).

### Intestinal manipulation and perioperative treatments

POI was induced by standardized intestinal manipulation as described previously^61^. Small bowel was eventrated after median laparotomy and gently rolled twice from oral to aboral using moist cotton. After reposition of the bowel the laparotomy was closed by a two layer suture. Some mice were pretreated with oral antibiotics (see below). In another experiment, wt animals received either two i.p. or i.v. injections of the antioxidant Edaravone (10 mg/kg bodyweight) or vehicle 30 min prior and 6 h after surgery. Edaravone was solved in 1 M NaOH and titrated with 1 M HCl to pH 7.2 before it was diluted in 0.9% saline for *in vivo* use. Control animals received injections of the same pH-titrated and saline-diluted mixture without Edaravone (=vehicle).

### Antibiotic treatment

According to Reikvam *et al*.^62^, some animals received oral gavages (200 µl) of the following antibiotics/antimycotics or normal drinking water (vehicle) for 7 days every 12 h (mg/kg bodyweight): vancomycin (50), gentamicin (100), metronidazole: (100), amphotericin-B (1). After 1 week stool samples were plated on Columbia blood agar plates and incubated for 48 h under aerobic or anaerobic condition. Bacterial growth was not detected after 7 days antibiosis. After the 7d treatment the animals underwent IM.

### *In vivo* gastrointestinal transit

Gastrointestinal transit (GIT) was measured by evaluation of the intestinal distribution of orally administered fluorescent labelled dextran-gavage (FITC-dextran, 70,000 MW, Sigma Aldrich) 90 minutes after administration as described previously^61^. The gastrointestinal tract was divided into 15 segments (stomach to colon). The geometric centre (GC) of labelled dextran distribution was calculated as described previously. The stomach (st) correlates with a GC of 1, the small bowel correlates with a GC of 2-11, the cecum (c) correlates with a GC of 12 and the colon (col) correlates with a GC of 13–15.

GIT-measurement was performed with naïve control (CTL) animals, 24 h and 72 h after IM.

### MPO^+^-cell infiltration

Jejunal mucosa-free muscularis whole mount specimen were fixed in ethanol and stained with Hanker Yates reagent (Polyscience Europe, Eppelheim, Germany) to identify Myeloperoxidase expressing cells (MPO^+^). A mean count of MPO+ cells/mm^2^ for 5 random areas per animal was determined. MPO^+^ measurement was performed with CTL animals, 24 h and 72 h after IM.

### Cell culture

Primary enteric glia cell cultures were obtained by sacrificing wt and Il1R1^−/−^ mice 8–16 weeks of age, extracting the small intestine and cleansing it with 20 ml of oxygenated Krebs buffer (126 mM NaCl; 2.5 mM KCl: 25 mM NaHCO_3_; 1.2 mM NaH_2_PO_4_; 1.2 mM MgCl_2_; 2.5 mM CaCl_2_, 100 IU/Penicillin, 100 IU/ml Streptomycin and 2.5 µg/ml Amphotericin). The small bowel was cut in 3–5 cm long segments and kept in oxygenated ice-cold Krebs buffer. Each segment was then put onto a sterile glass pipette and the muscularis externa was stripped with forceps to collect the muscle tissue for further digestion steps. After centrifugation (300 × g for 5 min), the tissue was incubated for 15 minutes in 5 ml DMEM containing Protease Type 1 (0.25 mg/ml, Sigma-Aldrich) and Collagenase A (1 mg/ml, Sigma-Aldrich) in a water bath at 37 °C with shaking. The digestion mix was stopped by adding 5 ml DMEM containing 10% FBS (Sigma-Aldrich), centrifuged for 5 min at 300 × g and resuspended in proliferation medium (neurobasal medium with 100 IU/Penicilin, 100 µg/ml Streptomycin, 2,5 µg/ml Amphotericin (all Thermo Scientific), FGF and EGF (both 20 ng/ml, Immunotools) in 37 °C, 5% CO_2_ for 4 days to form enteric neurospheres. For experiments, enteric neurospheres were dissociated with trypsin (0.05%, Thermo Scientific) for 5 min at 37 °C and distributed at a 50% confluency on Poly-Ornithin (Sigma-Aldrich) coated 6 well plates in differentiation medium (neurobasal medium with 100IU/Penicillin, 100 µg/ml Streptomycin, 2,5 µg/ml Amphotericin, B27, N2 (all Thermo Scientific) and EGF (2 ng/ml, Immunotools)). After 7 days in differentiation medium, the mature enteric glia cells were treated with IL-1α and IL-1β (10 ng/ml, Immunotools) for 6 h, washed with PBS and further processed for RNA isolation.

### Organ culture of ME and IL-1β ELISA

Release of IL-1β was measured by ELISA in organ culture supernatants of small bowel ME segments harvested 24 h after surgery. ME organ culture ME was performed in Dulbecco’s modified Eagle medium for 24 h at 37 °C, 5% CO_2_. Cell-free supernatants were assayed by ELISA for IL-1β (R&D Systems, Abingdon, England). Values were normalized to cultured tissue weights.

### Immunohistochemistry

Whole mount specimen were mechanically prepared by dissection of the (sub)mucosa, fixed in 4% paraformaldehyde/PBS for 30 min, permeabilized with 0.2% Triton-X 100/PBS for 15 min, blocked with 3% BSA/BPS for 1 h and incubated with primary IgGs (rabbit-IgG against ASC (Adipogen, 1:400), rat-IgG2a F4/80 (LifeTechnologies, 1:200) or rabbit-IgG anti 4-hydroxynonenal, abcam) at 4 °C overnight. After three PBS washing steps, secondary antibodies (Dianova, anti rat IgG-Cy3 1:1600 and anti-rabbit IgG-FITC or - Cy3 1:1600 were incubated for 90 min. Specimens were mounted in Fluorogel-Tris and imaged by a Nikon TE2000 microscope.

### FACS

Small bowel ME leukocytes were isolated from the ME after a 40 min incubation in 0.1% Collagenase type II (Worthington, New York, USA), 0.1 mg/mL DNase I (La Roche, Mannheim, Germany), 2.4 mg/mL Dispase II (La Roche), 1 mg/mL BSA and 0.7 mg/mL Trypsin inhibitor in HBSS a 37 °C. The cell suspension was filtered and single cells were analysed by flow cytometry. After an Fc-blocking step (clone 2.4G2), cells were stained for 30 min with antibodies (Biolegend) against CD45 (Pacific Blue, 30-F11), Ly6C (PE-Cy7, HK1.4), F4/80 (Al488, BM8) and Ly6G (APC, 1A8). For intracellular staining of IL-1β, cells were fixed and permeabilized with BD Cytofix/Cytoperm solution (BD Biosciences, Heidelberg, Germany) and incubated with an IL-1β antibody (PE, NJTEN3, eBioscience) for 30 min. Analyses were performed on a FACS Canto II (BD Biosystems, Heidelberg, Germany) and data were analysed with FlowJo (Tree Star Inc., Ashland, USA).

### RNA extraction and quantitative polymerase chain reaction

Total RNA was extracted from muscularis specimens at indicated time points after IM using the RNeasy Mini Kit (Qiagen, Hilden, Germany) followed by deoxyribonuclease I (Ambion, Austin, TX). Complementary DNA was synthesized using the high capacity cDNA reverse transcription kit (Applied Biosystems, Darmstadt, Germany). Expression of mRNA was quantified by real-time RT-PCR with SYBR Green QuantiTect Primer Assays (IL-1α, QT00113505; or TaqMan probes (IL-6, Mm00446190; IL-1β, Mm00434228; MCP-1, Mm00441242; Applied Biosystems) or primers against IL1R1 (forward 5′-CCTGCTCTGGTTTTCTTCCT-3′, reverse: 5′- CGGCAGTTTCTCCTTAGTGT-3′), IL1RII (forward 5′-TGCAAAGTGTTTCTGGGAAC-3′, reverse 5′-ATATTGCCCCCACAACCAAG-3′) IL1Rrap (forward 5′- TCGCATGGTATCTAGTCCCC-3′, reverse 5′-AAGGATGGGACTTCTGTGGT-3′).

Quantitative polymerase chain reaction was performed with SYBR Green PCR Master Mix (Applied Biosystems) or TaqMan Gene Expression Master Mix (Applied Biosystems).

### Detection of ROS, lipid peroxidation and oxidative stress-induced DNA damage

Oxidative stress was analysed by fluorescence microscopy (according to a modified protocol of Owusu-Ansah and colleagues^63^, with dihydroxyethidium (DHE, #12013, Biomol, Hamburg, Germany). Additionally, 4-hydroxy-noneal, a marker of lipid peroxidation, was detected by immunofluorescence microscopy after immunostaining with anti 4-hydroxynonenal antibody (1:200 dilution, abcam#46545). DNA modification was analysed a 8-hydroxy (OH)-2 deoxyguanosine ELISA (abcam # ab201734) in ME lysates following manufacturer’s instructions.

### Data analysis

Statistical analysis was performed with Prism V5.01 (GraphPad, San Diego, CA) using 1-way or 2-way analysis of variance with Bonferroni post-hoc or by Student t test as indicated. In all figures, p-value are indicated as *p < 0.05, **p < 0.01 and ***p < 0.001. All plots are show mean + standard errors.

## Supplementary information

Dataset 1
