# Author Correction: AIM2 inflammasome-derived IL-1β induces postoperative ileus in mice

**DOI:** 10.1038/s41598-020-60391-x

**Published:** 2020-02-21

**Authors:** Kristof Johannes Hupa, Kathy Stein, Reiner Schneider, Mariola Lysson, Bianca Schneiker, Veit Hornung, Eicke Latz, Yoichiro Iwakura, Jörg C. Kalff, Sven Wehner

**Affiliations:** 1 Department of Surgery, University of Bonn, Bonn, Germany; 2 Institute of Experimental Medicine, University of Bonn, Bonn, Germany; 3 Institute of Innate Immunity, University of Bonn, Bonn, Germany; 4 Research Institute for Biomedical Sciences, Tokyo University of Science, Chiba, 278-0022 Japan; 5 Present Address: Gene Center and Department of Biochemistry, Ludwig-Maximilians-Universität München, Munich, 81377 Germany

Correction to: *Scientific Reports* 10.1038/s41598-019-46968-1, published online 22 July 2019

The original version of this Article contained a typographical error in the spelling of the author Reiner Schneider, which was incorrectly given as Ralf Schneider. This has now been corrected in the PDF and HTML versions of the Article.

