# Supplementary material for: AIM2 inflammasome-derived IL-1β induces postoperative ileus in mice
Source: Sci Rep. 2019 Jul 22;9:10602. doi: 10.1038/s41598-019-46968-1 (PMC6646358; doi:10.1038/s41598-019-46968-1)
Supplement: Supplementary file 1 — Dataset 1 [file 41598_2019_46968_MOESM1_ESM.pdf]

Supplementary Information

AIM2 inflammasome-derived IL-1β induces postoperative ileus in mice

Hupa KJ[1], Stein K[1], Schneider R[1], Lysson M[1], Schneiker B[1], Hornung V[2,#], Latz E.[3], Iwakura Y[4], Kalff JC[1], Wehner S[1*].

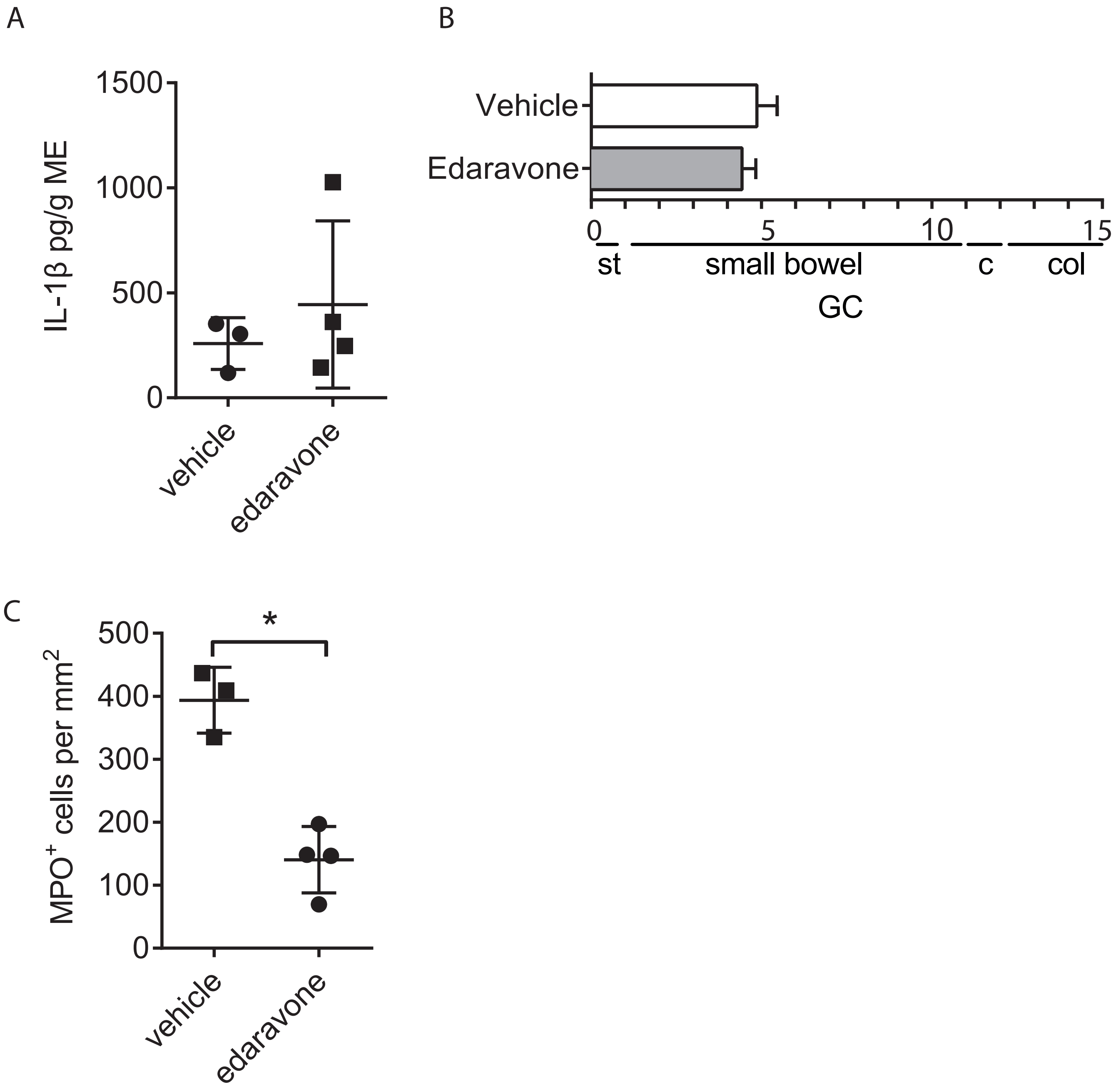

Supplementary figure S1
Wt mice underwent antioxidant (Edaravone) or vehicle i.v. injection and were analysed for (A) IL-1β release from ME organ cultures, (B) gastrointestinal motility and (C) numbers of myeloperoxidase (MPO)+ leukocytes 24h after IM. Statistical analyses: student´s t-test ($n_{vehicle}$=3, $n_{edaravone}$= 4). GC= geometric centre, st = stomach, c = cecum, col = colon
